# Energy expenditure in dogs before and after body weight reduction

**DOI:** 10.1186/1751-0147-57-S1-O19

**Published:** 2015-09-25

**Authors:** Caroline Larsson, Anne Vitger, Rasmus Bovbjerg Jensen, Peter Junghans, Anne-Helene Tauson

**Affiliations:** 1 Department of Veterinary Clinical and Animal Sciences, University of Copenhagen, Copenhagen, Denmark; 2 Department of Animal Nutrition and Management, Swedish University of Agricultural Sciences, Uppala, Sweden

## Introduction

Regain of body weight (BW) after a successful BW reduction program is a common problem. It has been discussed whether or not the maintenance energy requirement changes with reduced BW in dogs.

## Objective

The objective with this study was to investigate the impact of BW reduction and changes in body composition on the energy expenditure (EE) when measured under conditions corresponding to the basal metabolic rate (BMR).

## Material and methods

The EE in five privately owned, overweight dogs was measured by indirect calorimetry. Two measurements per dog were performed under the same standardised conditions (i.e. fasted and resting state) at the start, and after completing a 12-week BW reduction program including exercise and dietary restriction. Additionally, measurements of body composition by Dual-energy X-ray absorptiometry (DEXA) were conducted at the beginning and at the end of the BW reduction program.

## Results

The dogs lost 16% (SD ± 2.0) of their initial BW. Their fat mass was reduced (p<0.001), whereas fat free mass (FFM) remained unchanged. The EE decreased (p<0.001) with reduced BW from 4231 kJ/d at the start, to 3511 kJ/d at the end measurements. However, there was no effect of the BW reduction on the determined EE expressed in kJ/kg BW0.75/d, being 336 and 314 kJ/kg BW0.75/d at the start and end measurements, respectively.

## Conclusion

The results suggest that the BMR does not change with reduced BW in overweight dogs as long as the FFM remains unchanged.

